# Vascular rehabilitation interventions in people with peripheral arterial disease: an integrative review

**DOI:** 10.1590/1677-5449.202400882

**Published:** 2025-03-14

**Authors:** Kauane Flechas Arruda Perdigão, Larissa Pereira Costa, Annicia Lins Freitas, José Heriston de Morais Lima, Eduardo Ériko Tenório de França, Rafaela Pedrosa

**Affiliations:** 1 Universidade Federal da Paraíba – UFPB, João Pessoa, PB, Brasil.

**Keywords:** exercise therapy, peripheral arterial disease, rehabilitation

## Abstract

**Background:**

Peripheral artery disease, which occurs due to lower limb artery disorders, is associated with high cardiovascular mortality rates. Studies show that supervised exercise is an effective option for controlling symptoms.

**Objective:**

This study identified exercise types and complementary therapies used for vascular rehabilitation in people with peripheral artery disease and discusses the best recommendations in the literature.

**Methods:**

This integrative literature review is based on studies published in the last 5 years. The search was performed in the following databases: PubMed, SciELO, LILACS (BVS), and Cochrane. In addition to supervised exercise, the interventions in the clinical trials included other approaches that contributed to patient rehabilitation. Duplicate articles, articles whose full text was unavailable, and those whose title or abstract indicated they were unrelated to the topic were excluded.

**Results:**

Nine articles were included in the analysis. The results indicate that supervised exercise is the gold standard treatment method. However, therapies such as blood flow restriction, heat therapy, hydrotherapy, and resistance training can help improve treatment adherence, and their complementary effects benefit cardiovascular and physical function.

**Conclusions:**

In patients with peripheral artery disease, exercise-based rehabilitation is fundamental. However, resistance training with blood flow restriction can optimize muscle strength, while heat therapy and hydrotherapy can act as adjuvants to exercise.

## INTRODUCTION

Peripheral arterial obstructive disease, or peripheral arterial disease (PAD), which occurs due to systemic atherosclerotic processes that cause arterial obstructions, commonly affects the lower limbs. It can result in tissue ischemia, which varies according to the degree of arterial obstruction and the development of collateral circulation. In response to lower limb ischemia, several compensatory physiological processes are activated. The collateral arteries adapt to provide adequate blood supply to the lower limbs, and angiogenesis, ie, the emergence of new small arterial connections, can also occur^1,2^ .

This condition is associated with high rates of cardiovascular mortality. It is estimated that 10%-25% of the population over 55 years of age is affected by PAD, with the incidence increasing with age. Furthermore, approximately 70% to 80% of patients with the disease are asymptomatic. In 2013, the prevalence of PAD (asymptomatic or symptomatic) reported in high-income countries was similar between men and women and increased with age, rising from 5% in those aged 45–49 years to 18% in those aged 85-89 years^1,3^ .

One of the main symptoms in individuals affected by the disease is intermittent claudication, which includes pain and burning or cramping in the lower limbs during exercise, which is relieved by rest. Approximately one-third of people with the disease present these symptoms, in addition to pain resulting from ischemic neuropathy and pain at rest in more severe cases. Other symptoms include limb and muscle atrophy, ischemic ulcers, and gangrene, as well as dryness, scaling, and thickening of the skin and nails^3,4^ .

In addition to these consequences, PAD is associated with chronic changes in the morphology and function of the affected muscles. The main changes include muscle denervation, reduced nerve conduction velocity, selective atrophy of muscle fibers, and changes in enzyme activity. There is evidence that, together, these changes are associated with reduced muscle strength and worse functionality in patients with the disease. These studies also suggest that the observed decline in muscle strength may be influenced by sedentary lifestyle, which is frequent in these patients.^5^ Therefore, physiotherapeutic intervention is essential for symptom control.

There is consensus in the literature that supervised exercise is an effective option for symptom control and improved quality of life and prognosis in PAD. Treatment can also include pharmacological therapy and surgical procedures^4,6^ .

Thus, the objectives of this review were to identify exercise types and complementary therapies used in vascular rehabilitation for patients with PAD and to discuss the best recommendations in the literature.

## METHODS

This integrative literature review included studies published in the last 5 years to ensure that the most recent advances in the field are reflected and that the evidence aligns with technological, methodological, and conceptual changes. Among review types, integrative reviews involve the most comprehensive methodological approach because they allow the inclusion of both experimental and non-experimental studies, providing a more complete and in-depth understanding of the investigated phenomenon.^7^

The study was conducted in the following steps: identifying the theme; developing the research question; defining the inclusion and exclusion criteria; selecting the data to be extracted from the studies; searching for and selecting relevant studies; evaluating the included articles; extracting the data; analyzing and synthesizing the results; and, finally, presenting the data.

**Figure 1:**
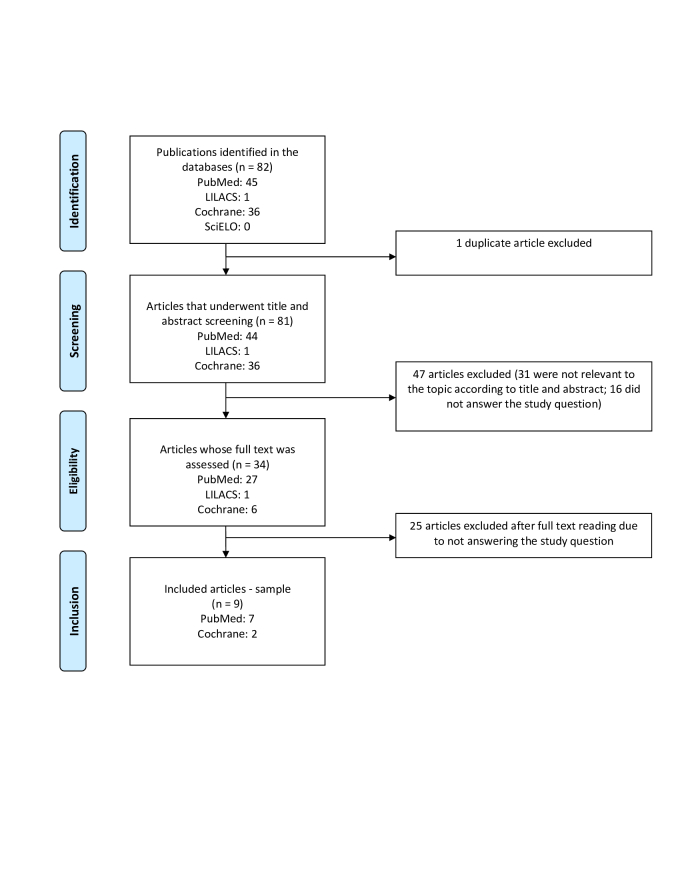
Flowchart for the selection of included studies - PRISMA model.

The following guiding question was used for studies that used exercise as a treatment for PAD: what additional therapies to supervised exercise have been found efficacious for PAD? To answer this question, a search was conducted in the following databases: MEDLINE/PubMed; Scientific Electronic Library Online; *Literatura Latino-Americana e do Caribe em Ciências da Saúde*, and the Cochrane database of systematic reviews. The keywords used in search strategy were: “exercise therapy”; “peripheral arterial disease”; “rehabilitation”. The combination of terms was adapted for each database, using the Boolean operator “AND” and filters for publication period, language, free full text and study types to be included (in this case, clinical trials). The search terms used were: (exercise therapy AND peripheral arterial disease AND rehabilitation). The identification, selection, eligibility, and inclusion of studies followed the Preferred Reporting Items for Systematic Reviews and Meta-Analyses guidelines, as shown in Figure 1.

The selected studies’ risk of bias was analyzed with the Revised Cochrane risk of bias tool for randomized trials (RoB 2.0), which uses the following 5 domains to identify bias in clinical trials: 1) bias in the randomization process, 2) deviations from the intended intervention, 3) bias due to missing data, 4) bias in measuring outcomes, and 5) bias in reporting outcomes. Additionally, the Oxford Centre for Evidence-Based Medicine tool for assessing levels of evidence (March 2009) was used to classify the quality and strength of evidence from clinical research, aiming to facilitate clinical decision-making.^8^

The following inclusion criteria were used: clinical trials published in the last 5 years; interventions in addition to supervised exercise or other approaches that contributed to the rehabilitation of patients with PAD; trials reporting the results of their interventions in terms of efficacy, safety, quality of life and/or functionality. Duplicate studies, those that did not meet the study objectives, those whose full text was not freely available and those whose title and/or abstract indicated they were unrelated to the topic were excluded.

## RESULTS

The search returned a total of 82 articles: 45 from PubMed, 1 from LILACS, 36 from Cochrane, and 0 from SciELO. One duplicate was identified. After screening the titles and abstracts, 47 articles were excluded, with the remaining 34 being selected for detailed analysis. At the end of the process, 9 studies were included, with a combined sample size of 629 participants (Table 1).

**Table 1:** Summary of original articles included in the review.

**Author**	**Level of evidence**	**Level of evidence according to the Oxford Centre for Evidence-Based Medicine (2009)** ^8^	**Objectives**	**Methods**	**Results**	**Conclusions**
McDermott et al.^9^	Domain 1: Low Risk	1a	To determine whether low-intensity home-based walking at a comfortable pace improves walking capacity in people with peripheral arterial disease vs high-intensity home-based walking exercise that induces ischemic leg symptoms and vs a no-exercise control protocol.	Randomized multicenter trial including 305 participants. The primary outcome was the mean change in distance walked in 6 minutes at 12 months.	Low-intensity exercise was significantly less effective in improving 6-min walk distance than high-intensity exercise. At the 12-month follow-up, there was no significant difference in change in 6-min walk distance between the low-intensity exercise group and the no-exercise control group.	High-intensity home exercise was more effective than low-intensity home exercise.
	Domain 2: Low Risk			Low-intensity walking exercise (n = 116), high-intensity walking exercise (n = 124), and no-exercise control (n = 65) for 12 months.		
	Domain 3: Low Risk			Exercise groups were asked to walk for exercise in an unsupervised environment, 5x per week, for up to 50 min per session, using an accelerometer to document exercise intensity and time.		
	Domain 4: Low Risk					
	Domain 5: Low Risk					
	Overview: Low Risk					
Akerman et al.^10^	Domain 1: Low Risk	1b	To test whether heat therapy is a viable alternative for patients with PAD.	Randomized, controlled clinical trial with two treatment groups, including a total of 22 participants.	After the interventions, total walking distance during the 6-minute walk test increased regardless of group. Pain-free walking distance increased. Systolic blood pressure reduced more after heat than after exercise, and diastolic blood pressure decreased by 4 mmHg in both groups. There were no significant changes in blood volume, ankle-brachial index, or measures of vascular health. There was no difference in improvement in functional or blood pressure outcomes between heat and exercise in individuals with PAD.	There was no difference in improvement in functional or blood pressure outcomes between heat and exercise in individuals with PAD.
	Domain 2: Low Risk			The heat group involved spa bathing at 39°C, 3-5 days/week, for ≤ 30 min, followed by ≤ 30 min of calisthenics. The exercise group involved ≤ 90 min of supervised walking and gym exercise, 1-2 days/week.		Heat therapy may improve functional capacity and has potential as an effective cardiovascular conditioning tool for individuals with PAD.
	Domain 3: Low Risk					
	Domain 4: Low Risk					
	Domain 5: Low Risk					
	Overview: Low Risk					
Parkington et al.^11^	Domain 1: Some concerns	2b	To evaluate the feasibility of a supervised blood flow restriction program in patients with claudication.	The study was a randomized controlled feasibility trial. The primary outcomes were the feasibility and acceptability of recruitment, allocation, measurement, and retention procedures.	Adherence to exercise was high. Clinical improvement in walking was achieved in 86% of patients in the BFR group but in only 46% of patients in the control group. Time to claudication pain during walking increased by 35% for the BFR group but remained unchanged for controls. Quality of life in the BFR group showed improvement in mobility, ability to perform usual activities, pain, depression, and general health at follow-up.	A supervised blood flow restriction program is feasible in patients with claudication and has the potential to increase exercise performance, reduce pain, and improve quality of life.
	Domain 2: Low risk			Thirty patients with stable claudication completed an 8-week supervised exercise program, randomized to receive either BFR (n = 15) or a combined exercise control without BFR (control, n = 15). The intervention consisted of a 5-min warm-up of light cycling with controlled cadence and load, followed by lower-body resistance exercises (leg press and knee extension). The exercises were performed bilaterally with repetitions every 3 seconds (1.5 s in the concentric phase and 1.5 s in the eccentric phase) with metronome support. Exercise for the BFR and control groups was combined with a relative volume load. Patients in the BFR group completed the resistance exercises with the addition of a pneumatic cuff.		
	Domain 3: Low risk					
	Domain 4: Low risk					
	Domain 5: Low risk					
	Overview: Some concerns					
	
Kapusta and Irzmański^12^	Domain 1: Some concerns	1b	To evaluate the effect of controlled physical training combined with hydromassage on changes in circuits, range of motion, and claudication distance in people with atherosclerotic ischemia of the lower limbs.	The study included 100 patients, men and women, aged between 39 and 79 years, with peripheral circulation disorders in the lower limbs.	A statistically significant reduction in foot, ankle, calf and thigh circumference was observed in group G. Both in groups G and CG, a statistically significant increase in the range of dorsiflexion of the foot was found. There was also a statistically significant increase in the range of plantar flexion movement of the foot.	Individually planned training, supplemented with hydrotherapy as a thermal therapy, has a beneficial effect on reducing swelling of the lower limbs, increasing the range of motion of the feet and extending the distance in the 6-minute walk test.
	Domain 2: Low risk			Experimental group: 10 hydromassage treatments in the lower limbs and individually prescribed training program. The exercises included breathing exercises, relaxation exercises and free active lower limbs, with toe raising and dorsal and plantar flexion. The hydromassage was performed using a rotating bathtub for the upper limbs, with a pressure of 2.5–3.5 kPa and a temperature of 39 ºC for 20 min.		
	Domain 3: Low risk			Control group: Training designed equally for all in the group.		
	Domain 4: Low risk					
	Domain 5: Low risk					
	Overview: Some concerns					
Bearne et al.^13^	Domain 1: Low Risk	1b	To investigate the effect of a home-based behavior change intervention of walking exercises delivered by physical therapists in adults with PAD and intermittent claudication compared with usual care.	The study was a multicenter, randomized, blinded, parallel-group clinical trial. The study included 19 adults with PAD. The outcomes were: 6-minute walk distance for 3 months, pain-free walking time, and maximal walking capacity. Participants were randomized to receive either a walking exercise behavior change intervention delivered by physical therapists trained to use a motivational approach (n = 95) or usual care (n = 95).	The 6-minute walk distance changed from 352.9 m at baseline to 380.6 m at 3 months in the intervention group and from 369.8 m to 372.1 m in the usual care group	Among adults with PAD and intermittent claudication, a home-based behavior change intervention of walking exercises (compared with usual care) resulted in improved walking distance at 3 months.
	Domain 2: Low Risk			The behavior change intervention used two psychological models and consisted of two 60-min face-to-face sessions and two 20-min telephone sessions over 3 months. The usual care group received no study intervention and received standard care provided by their vascular specialists.		
	Domain 3: Low Risk					
	Domain 4: Low Risk					
	Domain 5: Low Risk					
	Overview: Low Risk					
	
Paldán et al.^14^	Domain 1: Low Risk	2b	To assess changes in distance walked in the 6-minute walk test as the primary outcome measure. Secondary outcomes included changes in physical activity and assessment of patients' PAD-related quality of life.	The study consisted of a two-arm, blinded, randomized, controlled pilot trial including 39 participants. The primary outcome was change in 6-minute walk distance. The protocol was:	The average 6-minute walking distance increased in the intervention group, while the average distance decreased in the control group after 3 months of follow-up. PAD-related quality of life significantly increased in terms of “symptom perception” and “limitations in physical functioning”. User feedback showed increased motivation and a change in attitude towards supervised physical training.	In addition providing a valuable support tool for the user group, the TrackPAD mobile intervention was associated with a change in prognostically relevant outcome measures, combined with improved disease coping.
	Domain 2: Low Risk			Experimental group: routine care and the mobile intervention (TrackPAD) for the 3-month follow-up period.		
	Domain 3: Low Risk			Control group: routine care.		
	Domain 4: Low Risk					
	Domain 5: Low Risk					
	Overview: Low Risk					
Monteiro et al.^15^	Domain 1: Low Risk	1b	To compare the effects of two types of aerobic training (with and without lower limb load) on muscle metabolism in adults with PAD.	A randomized, single-blind clinical trial was conducted with 40 participants.	After the intervention, in both groups, there was a reduction in the relative recovery time, improvement in the reoxygenation rate, an increase in the resistance time after reaching the lowest muscle oxygen saturation, an increase in the distance covered, and improvement in walking economy in relation to StO_2_.	Traditional aerobic training was superior to modified training in improving muscle metabolism in patients with PAD.
	Domain 2: Some Concerns			Control group: traditional training with walking on the ground for 30 min, training on a treadmill without inclination at the average speed achieved during walking on the ground and then a progressive increase of 0.2 km/h.		
	Domain 3: Low Risk			Experimental group: training with progressive overload on the lower limbs, with the same exercises as the CG, but with added ankle weights and a gradual increase in load.		
	Domain 4: Low Risk					
	Domain 5: Low Risk					
	Overview: Some Concerns					
Villemur et al.^16^	Domain 1: Some concerns	1b	To assess the feasibility of a randomized clinical trial comparing parallel 4-week intensive rehabilitation programs comprising treadmill training performed as ITAR or conventional training with constant incline and speed interspersed with rest periods.	A randomized, prospective, blinded clinical trial was conducted with 38 participants. The primary outcome was change in maximum walking distance measured on a graded treadmill. The intervention consisted of:	Tolerance was excellent. Peak VO_2_ was low in both groups, corresponding to moderate-to-severe exercise intolerance. The two groups did not differ in the primary outcome or other walking distances (constant speed and graded treadmill test). For all 38 participants, both programs greatly increased maximal walking distance on the graded treadmill test.	An intensive 4-week rehabilitation program with ITAR or conventional training for intermittent claudication showed high adherence, was well tolerated, and improved walking distance as much as that reported for longer conventional programs.
	Domain 2: Low risk			in conventional training, subjects walked at a predetermined constant speed, stopping completely if they felt pain and resting until the pain subsided.		
	Domain 3: Low risk			In treadmill training performed with ITAR, subjects alternated between two different walking speeds and inclines, constituting the exercise and recovery periods. Each session lasted 40 minutes, including warm-up.		
	Domain 4: Low risk					
	Domain 5: Low risk					
	Overview: Some concerns					
Novaković et al.^17^	Domain 1: Low Risk	1b	To compare two types of supervised training (walking with moderate pain and without pain) with comparable intensity based on heart rate, in terms of walking capacity, quality of life, vascular function, biomarkers, and HRV in patients with intermittent claudication.	A randomized, open-label clinical trial was conducted with 36 adults, who were divided into 3 groups:	Walking capacity improved similarly in both training programs. Baseline walking distance and absolute walking distance increased significantly with moderate pain while walking. Quality of life also improved similarly in both training modalities, while only moderate pain while walking was also associated with a significant improvement in vascular parameters and flow-mediated vasodilation. Neither training program was associated with changes in biomarker levels or HRV.	Both pain-free and moderate-pain training modalities were safe and similarly improved walking capacity and health-related quality of life. In contrast, improvements in vascular function were only associated with moderate-pain walking.
	Domain 2: Low Risk			the pain training group walked on a treadmill until they felt moderate leg pain; they pedaled on a stationary bicycle for at least 5 min or until the leg pain disappeared;		
	Domain 3: Low Risk			the pain-free training group walked on a treadmill for two-thirds of the distance of the onset of claudication; the control group was advised to continue secondary preventive activities, including regular walking, as recommended by the treating vascular specialist.		
	Domain 4: Low Risk					
	Domain 5: Low Risk					
	Overview: Low Risk					

BFR: blood flow restriction; HRV: heart rate variability; ITAR: interval training with active recovery; PAD: peripheral artery disease.

In a comparative study between heat therapy vs supervised exercise therapy, Akerman et al.^10^ found no difference between the effects of heat therapy (through thermal baths) and a supervised exercise program. However, in cardiovascular adaptations related to blood pressure, all resting blood pressure variables were reduced in both interventions, but the decrease in systolic blood pressure was greater from the heat therapy intervention than the exercise intervention. However, it is important to emphasize that additional benefits, such as increased longitudinal strength and bone density changes, which are crucial for patients with PAD, will probably not be achieved through heat therapy alone.

Parkington et al.^11^ found that low-intensity resistance exercise with blood flow restriction was an important and effective rehabilitation tool for patients with claudication, since it can increase exercise performance, reduce pain, and optimize quality of life. Another effective method, discussed by Paldán et al.^14^ , was supervised physical activity supported by a smartphone application, since mobile health technology increases incentives and provides digital support at various levels of treatment.

Kapusta and Irzmański^12^ demonstrated that physical activity, complemented with hydrotherapy (such as thermal therapy), is an effective method for increasing exercise tolerance. They reported that this approach can reduce the need for more invasive and expensive interventions, since it provides significant cardiovascular and functional benefits in conservative treatment, especially when patients have exercise limitations.

Monteiro et al.^15^ found that traditional aerobic training was superior to modified walking training for improving muscle metabolism and optimizing muscle oxidative capacity and vascular function in patients with PAD. Furthermore, although improved deoxygenation rates were observed in both groups, more notable improvement occurred with traditional aerobic training, in addition to a clinically significant difference in gait economy compared to modified walking training.

McDermott et al.^9^ studied 3 groups, one that performed low-intensity walking exercises, another that performed high-intensity exercises, and a control group that did not exercise, finding significantly improved walking capacity in people with PAD. They found that low-intensity exercise was less effective than high-intensity exercise, and there were no significant walking performance results compared to the control group.

## DISCUSSION

According to the included studies, although supervised physical training is considered the gold standard for improving walking capacity and quality of life in patients with PAD, evidence suggests that alternative and complementary treatment methods may provide additional benefits. Some examples include a supervised program involving blood flow restriction, controlled physical training associated with hydromassage, a home-walking exercise program, and even interventions involving a smartphone app.

Studies have reported that supervised exercise therapy and standard care are effective rehabilitation methods for patients with PAD, despite evidence demonstrating low adherence to this type of intervention. In Akerman et al.^10^ , supervised exercise consisted of walking alone along a marked route for 30 minutes, followed by selected exercises in the hospital gym.

According to Abaraogu et al.,^18^ although supervised exercise training is a natural, effective, safe, economical, and compatible treatment for intermittent claudication and has a high level of evidence, its effectiveness, availability, access, and applicability are limited, as is patient adherence. A U.S. study by Criqui et al.^19^ found the following barriers to adherence to supervised exercise: 54% of physicians reported availability problems with facilities, as well as high treatment cost and a lack of professionals to supervise the exercise, while 49% of physicians reported that patients with PAD were not referred for exercise.

An alternative rehabilitation therapy for patients with PAD is resistance training, which can increase muscle endurance and strength. Over time, such training can result in an improved ability to climb stairs, higher self-assessment of physical function, greater physical fitness, and better quality of life, with adequate control of all movement variables (position and posture, range of motion, speed of execution, volume, and intensity). Resistance training may be appropriate as an adjunctive therapy to overground walking or treadmill walking exercise^18,20^ .

Furthermore, blood flow restriction is also mentioned as an additional means of enhancing the results of exercise. This procedure, performed with a pneumatic cuff, induces muscle and hemodynamic disturbances, activates the systemic production of hormones and the synthesis of myofibrillar and mitochondrial proteins, and promotes angiogenesis. These responses result in increased muscle strength and endurance, as well as improved physical function, including walking performance^11^ .

Heat therapy is another treatment option, with well-founded efficacy for cardiovascular conditioning. It has been demonstrated that this type of therapy provides a substantial antihypertensive stimulus, mainly by reducing systolic blood pressure, and this reduction is more significant with heat therapy than with exercise. However, when associated with exercise, heat therapy can increase functional improvement and cardiovascular conditioning.^21^ Kapusta e Irzmański^12^ corroborated this finding, demonstrating the beneficial effects of thermotherapy through hydrotherapy associated with systematic exercise, in that it improved foot range of motion, reduced edema, and increased claudication distance.

The present review offers significant contributions to vascular surgeons, given that they play a crucial role in patient referral and counseling. They must have extensive knowledge of treatment recommendations, since adherence to supervised exercise is often challenging for patients. Additionally, interventions such as hydrotherapy, thermotherapy, blood flow restriction exercise, and resistance exercise are complementary therapies that can further improve physical fitness and provide other significant benefits for people with PAD.

However, this review has certain limitations. First, the time window for publications and the number of included articles were limited, which may restrict the scope of the findings. In addition, few articles have been published on the topic, making a more comprehensive analysis difficult. Finally, we had difficulty accessing a relevant article from 2019, entitled “Peripheral Arterial Disease: Supervised Exercise Therapy Through Cardiac Rehabilitation”, which may have influenced diversity of information in the review.

## CONCLUSIONS

Although supervised exercise rehabilitation in patients with PAD is a fundamental therapeutic approach that offers numerous benefits, other methods have demonstrated efficacy in the rehabilitation process. Exercise training, especially supervised walking, effectively improves functional capacity, reducing symptoms of intermittent claudication and promoting overall cardiovascular health. Additionally, resistance training and blood flow restriction exercise can optimize muscle strength and functional capacity, while heat therapy and hydrotherapy can augment the beneficial effects of exercise, enhancing cardiovascular fitness and physical function.

In short, for patients with PAD, a combination of exercise types adapted to individual needs can result in significant improvement in quality of life and functional autonomy. Therefore, following diagnosis, it is important to perform a thorough assessment to identify the patient's needs and outline goals for controlling the disease. Nevertheless, more innovative means of raising awareness and better implementation are needed to reduce barriers, such as access, availability, and patient adherence, thus ensuring effective treatment and continued adherence to the rehabilitation program, which will maximize the benefits of exercise, improve patient quality of life, and reduce the risks associated with PAD.
