# Supplementary material for: Vascular rehabilitation interventions in people with peripheral arterial disease: an integrative review
Source: J Vasc Bras. 2025 Mar 14;24:e20240088. doi: 10.1590/1677-5449.202400882 (PMC11922327; doi:10.1590/1677-5449.202400882)
Supplement: Translation

# Translation: Intervenções para reabilitação vascular em pessoas com doenças arteriais obstrutivas periféricas: uma revisão integrativa

**DOI:** 10.1590/1677-5449.202400881

**Authors:** Kauane Flechas Arruda Perdigão, Larissa Pereira Costa, Annicia Lins Freitas, José Heriston de Morais Lima, Eduardo Ériko Tenório de França, Rafaela Pedrosa

**Affiliations:** 1 Universidade Federal da Paraíba – UFPB, João Pessoa, PB, Brasil.

**Keywords:** terapia por exercício, doença arterial periférica, reabilitação

## Abstract

**Contexto:**

A doença arterial obstrutiva periférica ocorre devido a afecções de artérias dos membros inferiores e está associada a elevadas taxas de mortalidade cardiovascular. Estudos mostram o exercício físico supervisionado como opção eficaz para o controle dos sintomas.

**Objetivos:**

Identificar quais são os tipos de exercícios e terapias complementares, adicionais ao exercício físico supervisionado, utilizados para a reabilitação vascular de pessoas com doença arterial obstrutiva periférica e discutir sobre as melhores recomendações da literatura.

**Métodos:**

Trata-se de uma revisão integrativa da literatura, construída baseada nas publicações dos últimos 5 anos. A busca ocorreu nas seguintes bases de dados: PubMed, SciELO, LILACS (Biblioteca Virtual da Saúde) e Cochrane. Foram incluídos ensaios clínicos dos últimos 5 anos, com intervenções adicionais ao exercício físico supervisionado ou outras abordagens que contribuíram para a reabilitação de pacientes. Foram excluídos artigos duplicados, sem texto completo disponível e sem informações relacionadas ao tema no título ou resumo.

**Resultados:**

Foram incluídos nove artigos para análise. Os resultados apresentados mostram que o exercício físico supervisionado é um método de tratamento considerado padrão-ouro. Entretanto, terapias como restrição de fluxo sanguíneo, terapia do calor, hidroterapia e treinamento resistido podem auxiliar na melhora da adesão do paciente, e seus efeitos complementares trazem benefícios cardiovasculares e para a função física. **Conclusões:** A reabilitação com exercício físico em pacientes com doença arterial obstrutiva periférica é uma abordagem terapêutica fundamental. Todavia, o treinamento de resistência com restrição de fluxo sanguíneo pode otimizar a força muscular, enquanto a terapia do calor e hidroterapia podem agir como coadjuvantes do exercício físico.

## INTRODUÇÃO

A doença arterial obstrutiva periférica (DAOP) ou doença arterial periférica (DAP) ocorre devido a afecções das artérias de membros inferiores, resultante de processos ateroscleróticos sistêmicos que culminam em obstruções arteriais e provocam isquemia dos tecidos, variando conforme o grau de obstrução arterial e o desenvolvimento da circulação colateral. Em resposta à isquemia dos membros inferiores, vários processos fisiológicos compensatórios são ativados. As artérias colaterais são adaptadas a fim de fornecer um suprimento sanguíneo adequado aos membros inferiores, além de ocorrer o processo de angiogênese, ou seja, o surgimento de novas pequenas conexões arteriais^1,2^ .

Essa condição está associada a elevadas taxas de mortalidade cardiovascular. Estima-se que entre 10 e 25% da população com mais de 55 anos seja afetada por essa obstrução, com a incidência aumentando conforme a idade avança. Além disso, aproximadamente 70 a 80% dos pacientes que possuem a doença são assintomáticos. Em países de alta renda, a prevalência da DAP (assintomática ou sintomática), relatada em 2013, foi semelhante entre homens e mulheres e aumentou com a idade, passando de 5%, entre 45 e 49 anos, para 18%, entre 85 e 89 anos^1,3^ .

Um dos principais sintomas manifestados por indivíduos acometidos pela doença é a claudicação intermitente, a qual consiste na presença de dor, queimação ou câimbra nos membros inferiores durante a prática de exercício físico, aliviada com o repouso. Aproximadamente um terço das pessoas com a doença apresenta essa sintomatologia, assim como a dor decorrente de neuropatia isquêmica e dor em repouso, em casos mais graves. Outros sintomas incluem atrofia do membro e da massa muscular, formação de úlceras isquêmicas e gangrenas, além de ressecamento, descamação e espessamento da pele e das unhas^3,4^ .

Além dessas consequências, a DAOP está associada a mudanças crônicas na morfologia e função da musculatura acometida. As principais alterações incluem desnervação muscular, redução da velocidade de condução nervosa, atrofia seletiva de fibras musculares e alterações na atividade enzimática. Há evidências indicando que, em conjunto, essas mudanças estão associadas a uma menor força muscular e pior funcionalidade em pacientes com a doença. Esses estudos sugerem também que o declínio observado na força muscular pode ser influenciado pelo sedentarismo frequente desses pacientes^5^ . Portanto, torna-se imprescindível a intervenção fisioterapêutica para o controle dos sintomas.

A literatura estabelece um consenso sobre os benefícios do exercício físico supervisionado como uma opção eficaz para o controle dos sintomas, melhoria da qualidade de vida e prognóstico dos pacientes com DAOP. Além disso, o tratamento pode incluir também a terapia farmacológica e procedimentos cirúrgicos^4,6^ .

Sendo assim, o objetivo do estudo foi identificar quais são os tipos de exercícios e terapias complementares, adicionais ao exercício físico supervisionado, utilizados para a reabilitação vascular de pessoas com DAOP e discutir sobre as melhores recomendações mencionadas na literatura.

## MÉTODOS

Trata-se de uma revisão integrativa da literatura, construída a partir de publicações realizadas nos últimos 5 anos, com o objetivo de garantir que as informações utilizadas na pesquisa reflitam os avanços mais recentes no campo de estudo e contribuam para a inclusão de dados e evidências alinhados com as mudanças tecnológicas, metodológicas e conceituais. A revisão integrativa destaca-se como a abordagem metodológica mais abrangente em comparação a outros tipos de revisões. Isso ocorre porque ela permite a inclusão tanto de estudos experimentais quanto não experimentais, proporcionando uma compreensão mais completa e aprofundada do fenômeno investigado^7^ .

O estudo foi conduzido através das seguintes etapas: identificação do tema; formulação da pergunta orientada da pesquisa; definição dos critérios de inclusão e exclusão dos artigos; seleção das informações a serem extraídas dos estudos; busca e seleção dos estudos relevantes; avaliação dos artigos incluídos; extração dos dados; análise e síntese dos resultados; e, por fim, apresentação dos dados.

Foram realizadas pesquisas de artigos que demonstrassem tratamento com exercício físico para a DAOP, utilizando a questão norteadora: quais terapias adicionais ao exercício físico supervisionado que demonstram eficácia no tratamento de pessoas com doenças arteriais obstrutivas periféricas? Para responder tal questionamento, foi realizada uma busca nas seguintes bases de dados: Publisher Medline (PubMed); Scientific Electronic Library Online (SciELO); Literatura Latino-Americana e do Caribe em Ciências da Saúde (LILACS) e a Cochrane. Os Descritores em Ciências da Saúde (DeCS) utilizados para estratégia de busca foram: “exercise therapy”; “peripheral arterial disease”; “rehabilitation”. A combinação de termos foi adaptada para cada base de dados específica, utilizando o operador booleano “AND” e filtros de período de publicação, idioma, texto completo gratuito e tipos de estudos a serem incluídos (ensaios clínicos). Dessa forma, a combinação dos termos de busca utilizados foram: (exercise therapy AND peripheral arterial disease AND rehabilitation). O processo de identificação, seleção, elegibilidade e inclusão dos estudos seguiu as diretrizes da declaração PRISMA (Preferred Reporting Items for Systematic Reviews and Meta-Analyses), como demonstrado na Figura 1.

**Figura 1:** Fluxograma de seleção de estudos incluídos - modelo PRISMA.

O risco de viés dos estudos selecionados foi analisado utilizando a ferramenta RoB 2.0 (*Revised Cochrane risk-of-bias tool for randomized trials*), que utiliza cinco domínios para identificar possíveis vieses nos ensaios clínicos. Os cinco domínios são: 1. viés no processo de randomização; 2. desvios da intervenção pretendida; 3. viés devido a dados faltantes; 4. viés na aferição dos desfechos e 5. viés no relato dos desfechos. Adicionalmente, foi utilizada a ferramenta Oxford Centre for Evidence-Based Medicine: níveis de evidência de março de 2009 para classificar a qualidade e força das evidências das pesquisas clínicas, visando facilitar a tomada de decisões clínicas^8^ .

Os critérios de inclusão dos estudos foram os seguintes: ensaios clínicos publicados nos últimos 5 anos; intervenções adicionais ao exercício físico supervisionado ou outras abordagens que contribuíram para a reabilitação de pacientes com DAP; ensaios que relatassem os resultados das intervenções em termos de eficácia, segurança, qualidade de vida e/ou funcionalidade dos pacientes.

Foram excluídos estudos duplicados, que não atenderam aos objetivos do estudo, que não tinham texto completo disponível gratuito e que não tinham informações relacionadas ao tema em seu título e/ou resumo.

## RESULTADOS

Foram encontrados 82 artigos na pesquisa realizada, dos quais 45 foram provenientes da PubMed, um da LILACS, 36 da Cochrane e nenhum da SciELO. Desses, um foi identificado como duplicata, estando disponível na PubMed e Cochrane. Após rastreamento dos artigos por meio do título e resumo, foram excluídos 47 artigos, sendo selecionados 34 estudos para análise detalhada. Ao final do processo, foram incluídos nove estudos, com número amostral de 629 participantes (Tabela 1).

**Tabela 1:** Resumo dos artigos originais incluídos na revisão.

**Autor**	**Nível de evidência**	**Nível de evidência pelo Oxford Centre for Evidence-Based Medicine (2009)** ^8^	**Objetivos**	**Métodos**	**Resultados**	**Conclusão**
McDermott et al.^9^	Domínio 1: Baixo risco	1a	Determinar se o exercício de caminhada domiciliar de baixa intensidade em um ritmo confortável melhora a capacidade de caminhada em pessoas com doença arterial periférica (DAP) *versus* exercícios de caminhada domiciliar de alta intensidade que induzem sintomas isquêmicos nas pernas e *versus* um controle sem exercício.	Ensaio multicêntrico randomizado. O estudo incluiu 305 participantes. O desfecho primário foi a mudança média na distância percorrida em 6miutos aos 12 meses.	O exercício de baixa intensidade foi significativamente menos eficaz na melhoria da distância percorrida em 6 min em comparação com exercícios de alta intensidade. No acompanhamento de 12 meses, não houve diferença significativa na mudança na distância percorrida em 6 minutos entre o grupo de exercício de baixa intensidade e o grupo controle sem exercício.	O exercício domiciliar de alta intensidade foi mais eficaz do que o domiciliar de baixa intensidade.
	Domínio 2: Baixo risco			Exercícios de caminhada de baixa intensidade (n = 116), exercícios de caminhada de alta intensidade (n = 124) e controle sem exercício (n = 65), por 12 meses.		
	Domínio 3: Baixo risco			Os grupos de exercício foram solicitados caminhar para se exercitar em ambiente não supervisionado, 5x por semana, por até 50 min por sessão, usando um acelerômetro para documentar a intensidade e o tempo do exercício.		
	Domínio 4: Baixo risco					
	Domínio 5: Baixo risco					
	Visão geral: Baixo risco					
Akerman et al.^10^	Domínio 1: Baixo risco	1b	Testar se a terapia térmica é uma terapia alternativa viável para pacientes com DAP.	Ensaio clínico randomizado e controlado, com dois grupos de tratamento. O estudo incluiu 22 participantes.	Após as intervenções, a distância total de caminhada durante um teste de caminhada de 6 minutos aumentou, independentemente do grupo. A distância de caminhada sem dor aumentou. A pressão arterial sistólica foi mais reduzida após o calor do que após o exercício e a pressão arterial diastólica diminuiu 4 mmHg em ambos os grupos. Não houve alterações significativas no volume sanguíneo, no índice tornozelo-braquial ou nas medidas de saúde vascular. Não houve diferença na melhora nos resultados funcionais ou de pressão arterial entre calor e exercício em indivíduos com DAP.	Não houve diferença na melhora nos resultados funcionais ou de pressão arterial entre calor e exercício em indivíduos com DAP.
	Domínio 2: Baixo risco			O grupo calor envolveu banho de spa a 39 ºC, 3-5 dias/semana, por ≤ 30 min, seguido de ≤ 30 min de exercícios calistênicos. O grupo exercício envolveu ≤ 90 min de caminhada supervisionada e exercícios na academia, 1-2 dias/semana.		A terapia térmica pode melhorar a capacidade funcional e tem potencial como uma ferramenta eficaz de condicionamento cardiovascular para indivíduos com DAP.
	Domínio 3: Baixo risco					
	Domínio 4: Baixo risco					
	Domínio 5: Baixo risco					
	Visão geral: Baixo risco					
		
Parkington et al.^11^	Domínio 1: Algumas preocupações	2b	Avaliar a viabilidade de um programa de restrição de fluxo sanguíneo (RFS) supervisionado em pacientes com claudicação.	O estudo consistiu em um ensaio de viabilidade controlado randomizado. Os resultados primários foram a viabilidade e aceitabilidade dos procedimentos de recrutamento, alocação, medição e retenção.	A adesão ao exercício foi alta. A melhora clínica na caminhada foi alcançada em 86% dos pacientes do grupo RFS, mas em apenas 46% dos pacientes do grupo controle. O tempo até a dor de claudicação durante a caminhada aumentou 35% para o RFS, mas permaneceu inalterado para o controle. A qualidade de vida do grupo RFS mostrou melhora na mobilidade, capacidade de realizar atividades habituais, dor, depressão e saúde geral no acompanhamento.	Um programa supervisionado de restrição do fluxo sanguíneo é viável em pacientes com claudicação e tem potencial para aumentar o desempenho nos exercícios, reduzir a dor e melhorar a qualidade de vida.
	Domínio 2: Baixo risco			30 pacientes com claudicação estável completaram um programa de exercícios supervisionados de 8 semanas, randomizados para receberem RFS (n = 15) ou um controle de exercício combinado sem RFS (controle, n = 15). A intervenção consistiu em aquecimento de 5 min de ciclismo leve, controlando cadência e carga; depois, foram realizados exercícios resistidos para parte inferior do corpo (*leg press* e extensão de joelhos). Os exercícios foram realizados bilateralmente com repetições executadas a cada 3 segundos (1,5 s na fase concêntrica e 1,5 s na fase excêntrica) com apoio de metrônomo. O exercício para os grupos RFS e controle foi combinado com uma carga relativa de volume. Os pacientes do grupo RFS completaram os exercícios resistidos com a adição de um manguito pneumático		
	Domínio 3: Baixo risco					
	Domínio 4: Baixo risco					
	Domínio 5: Baixo risco					
	Visão geral: Algumas preocupações					
	
Kapusta e Irzmański^12^	Domínio 1: Algumas preocupações	1b	Avaliar o efeito do treinamento físico controlado aliado às massagens com hidromassagem nas alterações dos circuitos, amplitude de movimento e distância de claudicação em pessoas com isquemia aterosclerótica de membros inferiores.	O estudo incluiu 100 pacientes, homens e mulheres, com idade entre 39 e 79 anos, com distúrbios da circulação periférica dos membros inferiores.	Observou-se redução estatisticamente significativa na circunferência do pé, tornozelo, panturrilha e coxa no grupo G. Tanto no grupo G quanto no GC foi encontrado aumento estatisticamente significativo na amplitude de dorsiflexão do pé. Houve também aumento estatisticamente significativo na amplitude de movimento de flexão plantar do pé.	O treino planejado individualmente, complementado com hidroterapia como terapia termal, tem um efeito benéfico na redução do inchaço dos MMII, no aumento da amplitude de movimentos dos pés e no prolongamento da distância no teste de caminhada de 6 minutos.
	Domínio 2: Baixo risco			Grupo experimental: 10 tratamentos de hidromassagem nos membros inferiores (MMII) e programa de treinamento prescrito individualmente. Os exercícios incluíram exercícios respiratórios, exercícios de relaxamento e MMII ativos livres, com subida dos dedos dos pés, flexões: dorsal e plantar. O banho de hidromassagem foi realizado por meio de uma banheira giratória para membros superiores, com pressão de 2,5–3,5 kPa e temperatura de 39 ºC durante 20 min.		
	Domínio 3: Baixo risco			Grupo controle: Treinamento elaborado igualmente para todos do grupo.		
	Domínio 4: Baixo risco					
	Domínio 5: Baixo risco					
	Visão geral: Algumas preocupações					
	
Bearne et al.^13^	Domínio 1: Baixo risco	1b	Investigar o efeito de uma intervenção domiciliar de mudança de comportamento de exercícios de caminhada realizada por fisioterapeutas em adultos com DAP e claudicação intermitente em comparação com os cuidados habituais.	O estudo foi um ensaio clínico randomizado, multicêntrico e cego para avaliados com dois grupos paralelos. O estudo incluiu 19 adultos com DAP. Os desfechos foram: a distância percorrida em 6 minutos de caminhada por 3 meses, o tempo de caminhada sem dor e a capacidade máxima de caminhada.	A distância caminhada de 6 minutos mudou de 352,9 m no início do estudo para 380,6 m aos 3 meses no grupo de intervenção e de 369,8 m para 372,1 m no grupo de cuidados habituais	Entre adultos com DAP e claudicação intermitente, uma intervenção domiciliar de mudança de comportamento de exercícios de caminhada, em comparação com os cuidados habituais, resultou em melhora da distância percorrida em 3 meses.
	Domínio 2: Baixo risco			Os participantes foram randomizados para receber uma intervenção de mudança de comportamento de exercício de caminhada realizada por fisioterapeutas treinados para usar uma abordagem motivacional (n = 95) ou cuidados habituais (n = 95).		
	Domínio 3: Baixo risco			Intervenção por mudança de comportamento usou dois modelos psicológicos e consistiu em duas sessões presenciais de 60 min e duas sessões telefônicas de 20 min, por 3 meses. O grupo de cuidados habituais não recebeu nenhuma intervenção do estudo e receberam tratamento padrão fornecido por seus especialistas vasculares.		
	Domínio 4: Baixo risco					
	Domínio 5: Baixo risco					
	Visão geral: Baixo risco					
	
Paldán et al.^14^	Domínio 1: Baixo risco	2b	Avaliar mudanças na distância percorrida no teste de caminhada de 6 minutos (metros) como medida de desfecho primário. Os desfechos secundários incluíram mudanças na atividade física e avaliação da qualidade de vida relacionada à DAP dos pacientes.	O estudo consistiu em um ensaio piloto de dois braços, cego, randomizado e controlado. Foram incluídos 39 participantes. O desfecho primário foi a mudança na distância de caminhada de 6 minutos. O protocolo foi:	O grupo intervenção aumentou a distância média de caminhada de 6 minutos, enquanto o grupo controle diminuiu a distância média após 3 meses de acompanhamento. A qualidade de vida relacionada à DAP aumentou significativamente em termos de “percepção de sintomas” e “limitações no funcionamento físico”. O *feedback* dos usuários mostrou maior motivação e uma mudança de atitude em relação à realização de treinamento físico supervisionado.	Além da classificação fornecer uma ferramenta de apoio valiosa para o grupo de usuários, a intervenção móvel TrackPAD foi associada a uma mudança nas medidas de resultados relevantes para o prognóstico, combinada com um melhor enfrentamento da doença.
	Domínio 2: Baixo risco			Grupo experimental: cuidados habituais e a intervenção móvel (TrackPAD) pelo período de acompanhamento de 3 meses.		
	Domínio 3: Baixo risco			Grupo controle: cuidados de rotina.		
	Domínio 4: Baixo risco					
	Domínio 5: Baixo risco					
	Visão geral: Baixo risco					
Monteiro et al.^15^	Domínio 1: Baixo risco	1b	Comparar os efeitos no metabolismo muscular de dois tipos de treinamento aeróbico, com e sem carga nos MMII, em adultos com DAP.	Foi realizado um ensaio clínico randomizado, simples e cego. Foram incluídos 40 participantes.	Após a intervenção, em ambos os grupos, houve redução do tempo relativo de recuperação, melhora na taxa de reoxigenação, aumento do tempo de resistência após atingir a menor saturação muscular de oxigênio, aumento da distância percorrida e melhora da economia da caminhada em relação à StO_2_	O treinamento aeróbico tradicional foi superior ao treinamento modificado em relação à melhora do metabolismo muscular em pacientes com DAP.
	Domínio 2: Algumas preocupações			Grupo controle: treino tradicional com caminhada no solo por 30 min, treinamento em esteira sem inclinação na velocidade média alcançada na caminhada solo e depois aumento progressivo de 0,2 km/h na velocidade.		
	Domínio 3: Baixo risco			Grupo experimental: treino com sobrecarga progressiva nos MMII, com os mesmos exercícios do GC, mas com acréscimo de caneleiras e aumento de carga gradativa.		
	Domínio 4: Baixo risco					
	Domínio 5: Baixo risco					
	Visão geral: Algumas preocupações					
	
Villemur et al.^16^	Domínio 1: Algumas preocupações	1b	Avaliar a viabilidade de um ensaio clínico randomizado comparando programas paralelos de reabilitação intensiva de 4 semanas, compreendendo treinamento em esteira realizado como treinamento intervalado com recuperação ativa (ITAR) ou treinamento convencional (TC) com inclinação e velocidade constantes intercalados com períodos de descanso.	Realizou-se um ensaio clínico randomizado, prospectivo, cego. O estudo incluiu 38 participantes. O desfecho primário foi a mudança da distância máxima de caminhada medida em uma esteira graduada. A intervenção consistiu em:	A tolerância foi excelente. O VO_2_ pico foi baixo em ambos os grupos, correspondendo a intolerância moderada a grave ao exercício. Os dois grupos não diferiram no desfecho primário ou outras distâncias de caminhada (velocidade constante e teste gradiente em esteira). Para todos os 38 participantes, ambos os programas aumentaram muito a distância máxima de caminhada no teste graduado em esteira.	Um programa intensivo de reabilitação de 4 semanas com ITAR ou TC para claudicação intermitente mostrou alta adesão, foi bem tolerado e melhorou a distância percorrida tanto quanto a relatada para programas convencionais mais longos.
	Domínio 2: Baixo risco			No treinamento convencional, os indivíduos caminharam em velocidade constante pré-determinada, parando completamente se sentissem dor e descansando até que a dor cessasse.		
	Domínio 3: Baixo risco			No treinamento com esteira realizado com ITAR, os indivíduos alternaram duas velocidades e inclinações de caminhada diferentes, constituindo os períodos de exercício e recuperação. Cada sessão durou 40 minutos, incluindo aquecimento.		
	Domínio 4: Baixo risco					
	Domínio 5: Baixo risco					
	Visão geral: Algumas preocupações					
Novaković et al.^17^	Domínio 1: Baixo risco	1b	Comparar dois tipos de treinamento supervisionado (caminhada com dor moderada e sem dor) com intensidade comparável com base na frequência cardíaca, em termos de capacidade de caminhada, qualidade de vida, função vascular, biomarcadores e variabilidade da frequência cardíaca (VFC) em pacientes com claudicação intermitente.	Realizou-se um ensaio clínico randomizado e aberto. O estudo incluiu 36 participantes adultos e eles foram divididos em três grupos:	Ambos os programas de treinamento melhoraram de forma semelhante a capacidade de caminhar. A distância inicial de caminhada e a distância absoluta de caminhada aumentaram significativamente com caminhada com dor moderada. A qualidade de vida também melhorou de forma semelhante com ambas as modalidades de treinamento, enquanto apenas a caminhada com dor moderada também foi associada a uma melhora estatisticamente significativa nos parâmetros vasculares, vasodilatação mediada por fluxo. Nenhum dos programas de treinamento foi associado a alterações nos níveis de biomarcadores e na VFC.	Ambas as modalidades de treinamento com dor moderada e sem dor foram seguras e melhoraram de forma semelhante a capacidade de caminhada e a qualidade de vida relacionada à saúde. Por outro lado, as melhorias na função vascular foram associadas apenas à caminhada com dor moderada.
	Domínio 2: Baixo risco			O grupo de treinamento com dor caminhou em uma esteira até sentir dor moderada nas pernas; pedalar em uma bicicleta ergométrica por pelo menos 5 min ou até o desaparecimento da dor nas pernas.		
	Domínio 3: Baixo risco			O grupo de treinamento sem dor caminhou em uma esteira até dois terços da distância do início da claudicação.		
	Domínio 4: Baixo risco			O grupo controle foi aconselhado a continuar com atividades preventivas secundárias, incluindo caminhada regular, conforme recomendado pelo especialista vascular responsável pelo tratamento.		
	Domínio 5: Baixo risco					
	Visão geral: Baixo risco					

RFS: restrição do fluxo sanguíneo; VFC: variabilidade da frequência cardíaca; ITAR: treinamento intervalado com recuperação ativa; DAP: doença arterial periférica.

Akerman et al.^10^ , em um estudo comparativo entre a terapia de calor *versus* a terapia de exercícios supervisionados, identificaram que não houve diferença evidente entre os efeitos da terapia térmica, por meio dos banhos termais, e um programa de exercícios supervisionados. Entretanto, nas adaptações cardiovasculares referentes à pressão arterial (PA), todas as variáveis da PA de repouso foram reduzidas em ambas as intervenções, porém a diminuição da pressão arterial sistólica (PAS) foi maior com o calor do que com o exercício. Todavia, é importante salientar que benefícios adicionais, como o aumento da força longitudinal ao longo dos ossos e as alterações resultantes na densidade óssea, que são cruciais para pacientes com DAP, provavelmente não serão alcançados apenas com a terapia de calor.

Parkington et al.^11^ , em seu estudo, trouxeram o exercício resistido de baixa intensidade com restrição de fluxo sanguíneo (RFS) como uma ferramenta de reabilitação importante e eficaz para pacientes com claudicação, pois esse possui a capacidade de aumentar o desempenho nos exercícios, diminuir a dor e otimizar a qualidade de vida. Outro método eficaz, trazido por Paldán et al.^14^ , foi a realização da atividade física supervisionada com suporte de um aplicativo para *smartphone*, uma vez que a tecnologia de saúde móvel aumenta os incentivos e fornece suporte digital em diversos níveis de tratamento.

Kapusta e Irzmańsk^12^ demonstraram em seu estudo que a atividade física, complementada com hidroterapia como terapia termal, é um método eficaz para aumentar a tolerância dos pacientes ao exercício. Além disso, os autores alegam que essa abordagem pode reduzir a necessidade de intervenções mais invasivas e dispendiosas, visto que proporciona benefícios cardiovasculares e funcionais significativos dentro do tratamento conservador, especialmente quando há limitação ao exercício.

Já Monteiro et al.^15^ apresentaram em seus estudos que o treinamento aeróbico tradicional apresentou superioridade em relação ao treinamento de caminhada modificado na melhoria do metabolismo muscular, otimizando tanto a capacidade oxidativa muscular quanto a função vascular em pacientes com DAP. Além disso, embora tenham sido observadas melhorias nas taxas de desoxigenação em ambos os grupos, o treinamento aeróbico tradicional demonstrou uma melhoria mais destacada nessas taxas, além de uma diferença clinicamente significativa na economia da marcha quando comparado ao treinamento de caminhada modificado.

McDermott et al.^9^ realizaram um estudo com três grupos, sendo um com exercícios de caminhada de baixa intensidade, outro de alta intensidade e um grupo controle sem realizar exercício, a fim de observar a melhora significativa da capacidade de caminhada em pessoas com DAP. Eles identificaram que o exercício de baixa intensidade foi menos eficaz do que o exercício de alta intensidade, além de não demonstrar resultados significativos para o desempenho da caminhada em comparação ao grupo controle sem exercício.

## DISCUSSÃO

De acordo com os resultados apresentados, apesar de o treinamento físico supervisionado ser um método de tratamento considerado padrão-ouro para melhora da capacidade de marcha e qualidade de vida em pacientes com DAP, as evidências apresentam outros métodos alternativos e complementares de tratamento. Alguns exemplos são o programa supervisionado com RFS, o treinamento físico controlado associado a hidromassagem, o exercício de caminhada domiciliar e até mesmo a inserção de uma intervenção por meio de um aplicativo de *smartphone*.

Em relação à terapia de exercício supervisionado e aos cuidados padrão, estudos os apresentam como métodos eficazes na reabilitação do paciente com DAP, apesar das evidências demonstrarem baixa adesão de indivíduos a esse tipo de intervenção. Akerman et al.^10^ , em seu estudo, demonstram que a execução do exercício supervisionado consiste em uma caminhada individualizada em um percurso marcado, durante 30 minutos, seguido de exercícios em circuitos selecionados na academia do hospital.

Abaraogu et al.^18^ trazem, em seu estudo, que apesar do treinamento de exercício supervisionado ser considerado um tratamento natural, eficaz, seguro, econômico e compatível com a diretrizes da claudicação intermitente, com alto nível de evidência, sua eficácia, disponibilidade, acesso e aplicabilidade são limitados, além de a adesão do paciente também ser restrita. Criqui et al.^19^ corroboram com o estudo e relatam barreiras de adesão aos exercícios supervisionados em um estudo realizado nos EUA. Entre as justificativas destacadas, observa-se que 54% dos médicos mencionaram a falta de disponibilidade de instalações, incluindo os custos associados a essas instalações e aos profissionais responsáveis pela condução dos exercícios. Adicionalmente, 49% dos médicos relataram a ausência de encaminhamentos dos pacientes com DAP para a prática dos exercícios.

Outrossim, uma terapia alternativa de reabilitação para pacientes com DAP é o treinamento de resistência, que contribui para a otimização da resistência muscular e da força dos indivíduos. Com o passar do tempo, esse treinamento pode resultar na melhoria da capacidade de subir escadas, da autoavaliação da função física, da aptidão física e da qualidade de vida desses indivíduos, com controle adequado de todas as variáveis do movimento (posição e postura, amplitude de movimento, velocidade de execução, volume e intensidade). O treinamento de resistência pode ser apropriado como terapia coadjuvante ao treinamento de caminhada no solo ou na esteira^18,20^ .

Além disso, a literatura apresenta a RFS como um método adicional para potencializar os resultados do exercício físico. Esse procedimento, realizado com um manguito pneumático, é considerado capaz de induzir perturbações musculares e hemodinâmicas, ativando a produção sistêmica de hormônios, a síntese de proteínas miofibrilares e mitocondriais, além de promover a angiogênese. Essas respostas resultam no aumento da força e resistência muscular, bem como na melhora da função física, incluindo o desempenho na caminhada^11^ .

Ademais, a terapia de calor constitui uma outra possibilidade de tratamento, considerando que sua eficácia para o condicionamento cardiovascular é bem fundamentada. Essa terapia demonstrou um estímulo anti-hipertensivo substancial, principalmente na redução da PAS, sendo essa redução mais significativa com a terapia de calor do que com o exercício físico. No entanto, é possível afirmar que a terapia térmica, quando associada ao exercício físico, pode potencializar a melhoria funcional e o condicionamento cardiovascular^21^ . O estudo de Kapusta e Irzmański^12^ corrobora com esse achado, pois traz os efeitos benéficos da termoterapia por meio da hidroterapia associado a exercícios sistemáticos, apresentando melhoria da amplitude de movimento, diminuição do edema e aumento da distância de claudicação.

Portanto, este estudo oferece contribuições significativas aos especialistas em cirurgia vascular, dado que esses profissionais desempenham um papel crucial no encaminhamento e orientação dos pacientes. É essencial que seu extenso conhecimento sobre as recomendações de tratamento esteja bem delineado, uma vez que a adesão ao exercício físico supervisionado frequentemente apresenta desafios para os pacientes. Adicionalmente, intervenções como hidroterapia, termoterapia, exercícios com RFS e exercícios resistidos representam terapias complementares ao exercício físico, proporcionando melhorias na aptidão física e outros benefícios potencializadores significativos ao paciente com DAP.

No entanto, o presente estudo apresenta algumas limitações. Primeiramente, o intervalo de tempo considerado para os artigos analisados e a quantidade de artigos selecionados foram limitados, o que pode restringir a abrangência dos achados. Além disso, houve uma escassez de artigos publicados sobre o tema, dificultando uma análise mais abrangente. Por fim, enfrentou-se a dificuldade de acesso a um artigo relevante, intitulado como “Peripheral Arterial Disease: Supervised Exercise Therapy Through Cardiac Rehabilitation” e publicado em 2019, o que pode ter influenciado na carência da diversidade de informações da revisão.

## CONCLUSÃO

A reabilitação com exercício físico supervisionado em pacientes com DAP é uma abordagem terapêutica fundamental que oferece inúmeros benefícios. Todavia, outros métodos também demonstram eficácia no processo de reabilitação. O treinamento físico, especialmente a caminhada supervisionada, tem se mostrado eficaz na melhoria da capacidade funcional, na redução dos sintomas de claudicação intermitente e na promoção da saúde cardiovascular geral. Adicionalmente, a inclusão de treinamento de resistência e com RFS pode otimizar a força muscular e a capacidade funcional, enquanto a terapia de calor e a hidroterapia podem complementar os efeitos benéficos do exercício físico, potencializando o condicionamento cardiovascular e a função física.

Em suma, a combinação de diferentes modalidades de exercício físico, quando adaptadas às necessidades individuais dos pacientes, pode proporcionar uma melhora significativa na qualidade de vida e na autonomia funcional dos indivíduos com DAOP. Por isso, é importante que, após o diagnóstico, seja realizada uma avaliação minuciosa para identificar as necessidades do paciente e traçar objetivos que busquem o controle da doença. Ainda assim, é necessário que sejam realizadas conscientização e implementação mais inovadoras para reduzir as barreiras como acesso, disponibilidade e adesão do paciente, a fim de garantir a eficácia do tratamento e a adesão contínua ao programa de reabilitação. Dessa maneira, será possível maximizar os benefícios do exercício físico, melhorar a qualidade de vida dos pacientes e reduzir os riscos associados à DAOP.
